# A rare case report of diverticulum of the buccal mucosa

**DOI:** 10.1002/ccr3.7566

**Published:** 2023-07-05

**Authors:** Kazushige Koike, Hinako Mori, Kazuhiro Ogi, Makiya Jin, Satoshi Ohwada, Takahiro Iwamoto, Shintaro Sugita, Tadashi Hasegawa, Akihiro Miyazaki

**Affiliations:** ^1^ Department of Oral Surgery Sapporo Medical University School of Medicine Sapporo Japan; ^2^ Department of Surgical Pathology Sapporo Medical University School of Medicine Sapporo Japan

**Keywords:** buccal mucosa, diverticulum, oral cavity, oral pathology

## Abstract

We report a case of diverticulum of the buccal mucosa. A 56‐year‐old man had a small pouch‐shaped lesion behind the parotid papilla that caused pain and food impaction. After resection, the lesion was histopathologically diagnosed as diverticulum without buccal muscle tear. There has been no recurrence during 1 year postoperatively.

Diverticulum is common in the colon and other areas of the gastrointestinal tract, and is generally cystic in shape, with a portion of the wall of the gastrointestinal tract protruding outward. It is thought to be caused by increased internal pressure and vulnerability of the gastrointestinal tract wall.^1^ The most common type of diverticulum affecting the colon is pseudodiverticulum, in which the sac‐like mucosa herniates or projects through the muscularis propria.^2^ Most cases are asymptomatic, and the prevalence increases with age, becoming particularly common in people over 80 years old.^3^ Diverticulum in the oral cavity is rare, with only about 15 cases reported.^4^, ^5^, ^6^, ^7^, ^8^, ^9^, ^10^, ^11^, ^12^, ^13^, ^14^, ^15^, ^16^

A 56‐year‐old Japanese man was referred to our hospital with pain in the right buccal mucosa. He had previously been diagnosed with stomatitis at a family dental clinic, which was treated with corticosteroid ointment. However, his symptoms had not improved. Intraoral examination revealed a pouch‐shaped depression about 4 mm in diameter and 6 mm deep behind the right parotid papilla (Figure 1A,B). Differential diagnosis could include salivary gland tumor, cystic lesion, or diverticulum, which required imaging studies. Contrast medium‐soaked gauze was placed in the oral cavity and enhanced computed tomography was performed. An enhancing area measuring 5 mm in size and 9 mm in depth was seen 20 mm dorsal to the orifice of the right parotid duct and extending under the buccal mucosa (Figure 2A,B). Magnetic resonance imaging revealed an oval nodule measuring 7 × 3 mm in size and 5 mm in depth with poor contrast enhancement under the buccal mucosa 20 mm dorsal to the right parotid orifice; the lesion showed no signal on a T1‐weighted image (Figure 2C) and low signal on a T2‐weighted image (Figure 2D). Ultrasonography showed a hypoechoic area measuring about 4 × 3 mm in size and 3 mm in depth in the right buccal mucosa that had a diverticulum‐like appearance. The interior of the pouch‐shaped lesion was isoechoic to hypoechoic. Color Doppler examination did not show an increased blood flow signal in the lesion (Figure 2E,F). Based on the clinical and imaging findings, the diagnosis was diverticulum of the buccal mucosa. When the tip of a syringe was inserted into the depression and rinsed with saline, a white substance that appeared to be food residue flowed out. Thereafter, the patient's pain resolved. However, he continued to complain of residual discomfort and impaction of food residue. This was attributed to a wisdom tooth, which made it difficult to clean the area and remove food particles. We conducted wisdom tooth extraction in order to resolve his complaints. Nevertheless, his complaints did not resolve completely after extraction of the wisdom tooth, so we surgically resected the lesion under general anesthesia. An incision line was set around the depression, and the pouch‐shaped lesion was excised and removed intact. The mucosa was sutured after confirming that there was no obvious buccal muscle tear. Histopathological examination of the hematoxylin–eosin‐stained specimen showed a cystic structure lined by stratified squamous epithelium extending from the subepithelial connective tissue to the fatty tissue in the buccal mucosa and covered by stratified squamous epithelium (Figure 3A,B). The findings were consistent with a diverticulum. The patient has been asymptomatic with no evidence of recurrence as of 1 year postoperatively.

**FIGURE 1:**
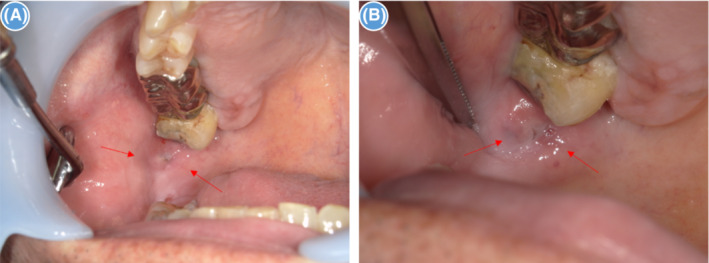
Intraoral views. (A): Intraoral photograph showing a pouch‐shaped depression about 4 mm in diameter and 6 mm in depth behind the right parotid papilla (arrowhead). (B): Enlarged image (arrowhead).

**FIGURE 2:**
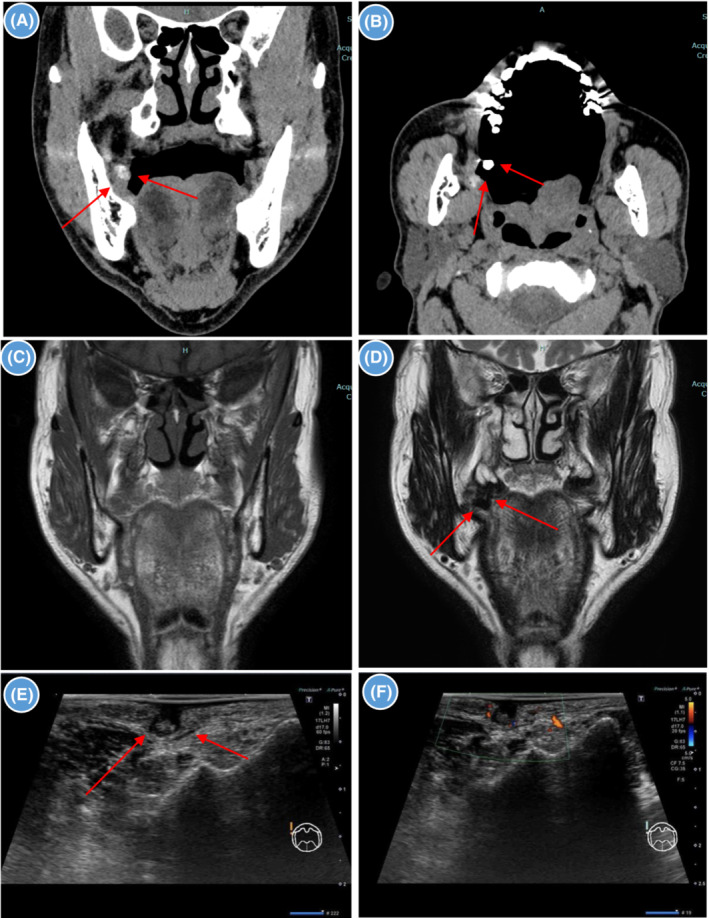
Radiological images. (A, B): Contrast‐enhanced computed tomography images showing an area of contrast under the buccal mucosa (arrowhead). (C, D): Magnetic resonance images showing an oval nodule with a poor contrast effect under the buccal mucosa; there is no signal on the T1‐weighted image and low signal on the T2‐weighted image (arrowhead). (E, F): An ultrasound scan showing a hypoechoic mass in the right side of the buccal mucosa. The interior of the mass is isoechoic to hypoechocic (arrowhead). Color Doppler examination did not reveal increased blood flow signal in the mass.

**FIGURE 3:**
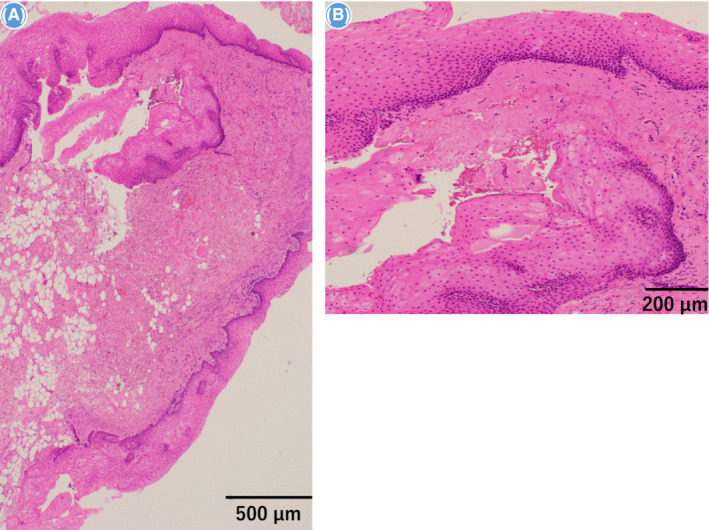
(A, B): Histopathological findings in the resected tissue specimen. Hematoxylin–eosin staining showed a cystic structure lined by stratified squamous epithelium extending from the subepithelial connective tissue to the fatty tissue in the buccal mucosa and covered with stratified squamous epithelium.

Diverticula are classified as congenital or acquired, unilateral or bilateral, and true or pseudo. A true diverticulum involves all layers of the structure, including the muscularis propria and adventitia, whereas a pseudodiverticulum does not involve the muscular layers or adventitia. In this case, considering that there were no findings of rupture of the muscular layer, the diverticulum was considered to be an acquired unilateral pseudodiverticulum.

Diverticulum has four possible causes. The first is a defect arising from dysplasia of the buccal muscles; anatomically, the cheek is composed mainly of the buccinator muscle, which consists of three groups of muscle fiber. It has been proposed that a diverticular lesion is caused by a developmental anomaly involving partial dissection along the buccinator muscle fibers.^10^, ^11^ The second cause is prolonged food impaction, which leads to compression and atrophy of the mucosa and muscles, resulting in a concave depression. Rowson^14^ postulated that chronic food impaction causes a small traumatic lesion that might progress to a diverticulum over time as a result of thin atrophic mucosa and poor muscle tone. Kubo et al.^15^ also suggested the possibility of a relationship with chronic food impaction in view of the prevalence of this condition in the elderly, in whom poor oral hygiene is common. The third cause is necrosis of a salivary gland neoplasm. Bailey^16^ postulated that formation of a diverticulum may result from an abnormal growth of salivary tissue. Hypothetically, a salivary gland neoplasm arising from aberrant salivary tissue may become necrotic and subsequently form a diverticulum in the normal squamous buccal mucosa. The final possible cause is a developmental abnormality of the buccal mucosa or aborted development of accessory parotid primordial invaginations. It has been suggested that diverticulum of the buccal mucosa is a consequence of idiopathic developmental defects resulting from invagination of the primary epithelial band, or may represent aborted development of parotid or accessory parotid primordial invaginations^12^ or of buccal glands.^17^ In our case, the diverticulum may have occurred because of the presence of a wisdom tooth, which made the area difficult to clean, leading to retention of food residue and persistent inflammation.

Histopathologically, the epithelial lining of the diverticulum is continuous with the surrounding buccal mucosa and the subepithelial connective tissue is accompanied by variable amounts of inflammatory infiltrate. The muscular tissue below the connective tissue may not be continuously present.^4^, ^5^

Most diverticula of the gastrointestinal tract diverticula do not require intervention if they are asymptomatic. In terms of treatment, surgery has often been performed for an oral diverticulum to address food impaction and the associated inflammation and to perform differential diagnosis.^4^, ^5^, ^6^, ^7^, ^9^, ^10^, ^12^, ^16^ However, resection should be limited to cases in which there are symptoms such as swelling and impaction of food debris. In cases where surgery has been performed, no recurrence has been observed. However, if the diverticulum is asymptomatic and the patient has an age‐related organic or functional disorder, such as stroke and dysphagia, conservative follow‐up may be more appropriate.^10^, ^12^ If food impaction is present, oral hygiene measures may be warranted to prevent inflammation. Enlargement of the diverticulum during follow‐up has been reported,^16^ so clinicians should monitor such cases carefully. Also, it is always necessary to perform the histopathological examination in all the removed lesions of the oral cavity in order to ensure diagnosis accurately.^18^

This is a rare report of a case of diverticulum of the buccal mucosa that was confirmed by clinicopathological findings and computed tomography, magnetic resonance imaging and ultrasonography. We hope that this report will assist clinicians in differential diagnosis of lesions in the oral cavity.

## AUTHOR CONTRIBUTIONS

**Kazushige Koike:** Conceptualization; formal analysis; investigation; resources; writing – original draft; writing – review and editing. **Hinako Mori:** Investigation; resources; writing – review and editing. **Kazuhiro Ogi:** Resources; writing – review and editing. **Makiya Jin:** Resources; writing – review and editing. **Satoshi Ohwada:** Resources; writing – review and editing. **Takahiro Iwamoto:** Resources; writing – review and editing. **Shintaro Sugita:** Resources; supervision; writing – review and editing. **Tadashi Hasegawa:** Supervision; writing – review and editing. **Akihiro Miyazaki:** Conceptualization; supervision; writing – review and editing.

## FUNDING INFORMATION

The authors received no financial support for the research, authorship, and/or publication of this article.

## CONFLICT OF INTEREST STATEMENT

The authors declared no potential conflicts of interest with respect to the research, authorship, and/or publication of this article.

## ETHICS STATEMENT

The study was performed in accordance with the principles of the Declaration of Helsinki. The need for ethical approval was waived by institution as this is not required for case reports.

## CONSENT STATEMENT

The patient provided written informed consent for the publication of this report and use of his image.

## Data Availability

All relevant data are within the paper.
